# Differences in category information processing between areas TEO and TE of the macaque

**DOI:** 10.3389/fnbeh.2024.1449097

**Published:** 2025-01-29

**Authors:** Masaumi Shimizu, Shun Katakami, Masato Okada, Yasuko Sugase-Miyamoto, Kazuko Hayashi, Keiji Matsuda, Kenichiro Miura, Mark A. G. Eldridge, Richard C. Saunders, Barry J. Richmond, Narihisa Matsumoto

**Affiliations:** ^1^Graduate School of Frontier Sciences, The University of Tokyo, Kashiwa, Japan; ^2^Human Informatics and Interaction Research Institute, National Institute of Advanced Industrial Science and Technology (AIST), Tsukuba, Japan; ^3^Research Fellow of Japan Society for the Promotion of Science, Chiyoda, Tokyo, Japan; ^4^Department of Pathlogy of Mental Diseases, National Center of Neurology and Psychiatry, National Institute of Mental Health, Kodaira, Tokyo, Japan; ^5^Section of Brain Function Information, National Institute for Physiological Sciences, Okazaki, Japan; ^6^Laboratory of Neuropsychology, National Institute of Mental Health, Bethesda, MD, United States

**Keywords:** visual categorization, category information processing, inferior temporal cortex, logistic regression, linear discrimination analysis

## Abstract

Object categorization is a fundamental visual function, via which primates group items based on perceptual similarity. Neurons that respond to a class of complex objects, such as faces, can be found in inferior temporal cortex of macaque monkeys, comprising areas TEO and TE. The ability of monkeys to categorize cat/dog images is greatly impaired when both TE and TEO are removed, but is only modestly impaired if either region is left intact. This suggests that both TE and TEO can support object categorization. We investigated what differences exist in category information processing between areas TEO and TE. For cat and dog stimulus images, we found that category decoding performance increased during the initial phase of a stimulus presentation, then remained stable in area TEO for the duration of the presentation in a passive fixation task. In area TE, category decoding performance continued to improve into later in the time window than in TEO. Furthermore, we found that, after cat/dog category training, area TE neuronal populations encode cat and dog category information more strongly than do TEO neurons even in a fixation task (Mann-Whitney U-test, *p* < 0.05). Together, our results suggest that area TEO processes category information without changing its representation, whereas the category information representation in area TE evolves over time (both within a trial and across category training sessions), indicating that responses in TE may be influenced by top-down feedback.

## 1 Introduction

Primates, such as humans and monkeys, possess the ability to instantaneously categorize objects based on their visual characteristics. The visual information relating to object categorization is thought to be processed through the ventral stream (Mishkin et al., 1983). The ventral stream is a hierarchical sequence of visual areas, comprising the primary visual cortex V1, V2, V4, and the inferior temporal cortex (IT), comprising areas TEO and TE. Broadly speaking, V1 processes object contours, V2 processes properties of object surfaces, V4 processes information about color and shape, and IT integrates the information from the earlier stages (Desimone and Gross, 1979; Mishkin et al., 1983; Murray et al., 2007). Within IT, neurons in area TE have larger receptive fields than those in area TEO, with the former responding to whole faces and the latter to facial parts, due to the differences in receptive field size and feature selectivity (Kobatake and Tanaka, 1994).

Electrophysiological experiments have extensively studied the correlation between TE neuron activity and categorization, revealing that TE contains neurons selectively responsive to various visual perceptual categories (Gross et al., 1972; Rolls, 1984; Tanaka et al., 1991; Fujita et al., 1992). Research on neuronal population representation of categories has found that activity patterns in TE neurons can encode object category information (Kiani et al., 2007; Meyers et al., 2008; Kriegeskorte et al., 2008; Pearl et al., 2024). The temporal dynamics of information representation encoded by neurons in area TE remain under debate. Neurons in area TE encode coarse-level categorization (e.g., human vs. monkey faces) in the early phase of the visual response and fine-level categorization (e.g., individual faces) in the later phase (Sugase et al., 1999; Matsumoto et al., 2005). Studies using larger image sets have indicated that neuron populations in area TE represent mid-level categories (e.g., human faces) earlier than superordinate (e.g., animate vs. inanimate) or subordinate (e.g., individual faces) levels (Dehaqani et al., 2016).

Behaviorally, the bilateral removal of areas TEO or TE causes only mild impairments (Matsumoto et al., 2016; Eldridge et al., 2018; Setogawa et al., 2021), but simultaneous removal of both areas results in significant deficits (Setogawa et al., 2021). On the other hand, removal of TEO produces no impairments in visual memory-dependent behavioral tasks, while removal of TE results in significant deficits (Eldridge et al., 2023).

These behavioral experimental results suggest that both areas TEO and TE play important roles in processing category information and that there are functional differences between the two areas. However, electrophysiological experiments often do not distinguish between TEO and TE, or only record from TE, leading to a limited understanding of the differences in neuronal characteristics between TEO and TE. Kobatake and Tanaka (1994) showed TEO neurons responded to parts of faces, and TE neurons responded to whole faces, but they did not investigate whether TE and TEO neurons changed their responses before and after visual category training. Furthermore, the results of Sugase et al. (1999) and Matsumoto et al. (2005) that category information encoded by TE neuronal population changed temporally indicate a possibility that category information encoded by TE and TEO neuronal population have different temporal changes. Pearl et al. (2024) recorded neuronal activity in TE of two monkeys performing a passive fixation task before and after category training. The study revealed that the ability of TE neurons to discriminate between categories improved during the presentation of images. In this study, we recorded the neuronal activity in TE from three monkeys (including the two reported in Pearl et al. (2024)), and in TEO from two of the three, while they performed a passive fixation task before and after category training by implanted Utah arrays. We analyzed category information encoded by neuronal populations of each area using linear classifiers (logistic regression model). Neuronal population in area TEO demonstrated temporally stable categorization capabilities after category training. In other words, categorization accuracy did not improve after category training. Neuronal populations in area TE showed improved categorization accuracy after category training.

## 2 Materials and methods

### 2.1 Experimental procedure

#### 2.1.1 Passive fixation task

Detailed experimental procedures have been reported previously (Pearl et al., 2024). Three male monkeys [Monkey X (weight: 11 kg, age: 13 years old), Monkey R (9kg, 12 years old), and Monkey L (9 kg, 8 years old)] (*Macaca fuscata*) performed a passive fixation task while seated in a primate chair positioned in front of a monitor on which visual stimuli were displayed. In the fixation task, a trial began when a blue target (size: 0.4 degrees x 0.4 degrees) appeared in the center of the monitor. After the monkey fixated on the blue target for 200–300 ms, five visual stimuli were randomly chosen and presented behind the blue target each for 350–400 ms, with a 350–400 ms inter-stimulus interval. If the monkey maintained fixation until the end of the trial, the blue target disappeared, and water drops were delivered as a reward. After a one-second inter-trial-interval, the next trial began with a new set of five stimuli. In this study, we focused whether the category training changed the responses of TE or TEO neurons. In the previous ablation studies of Matsumoto et al. (2016); Eldridge et al. (2018); Setogawa et al. (2021) cat and dog images were used. Therefore, we use the same cat and dog images to compare the previous studies. The visual stimuli consisted of colored pictures of 260 cats and 260 dogs (size: 12 degrees x 12 degrees). Using a passive fixation task reduces the confounds from motor planning and execution, and of reward biases, allowing us to examine the influence of visual categories alone.

We recorded 2 days of baseline passive fixation task for Monkey X, 6 days for Monkey R, and 4 days for Monkey L. The number of total correct trials in all baseline sessions was nearly equal for each monkey (approximately 4,000 correct trials). After these baseline sessions, the monkeys performed the passive fixation task with various cat/dog images for 2–3 months. We recorded one pre-training passive fixation session before cat or dog category training for all monkeys. After the 3–8 category training sessions, we recorded post-training 2 days of passive fixation task for Monkey R—because the first day had too low a trial count only data from the second day was used for analysis—and 1 day for Monkeys X and L.

#### 2.1.2 Category training

In the category training task (Eldridge et al., 2018), a trial began when the monkey touched a bar, and an image of a dog or cat was presented. After 350–400 ms, a red target appeared in the center of the monitor, and after 1–3 seconds, it turned green. If the monkey released the bar on red, the trial ended, whereas if the monkey released the bar on green, the trial outcome was delivered. The trial outcome was either a reward or a timeout (4–6 seconds), depending on the category of the image. Dog images were associated with a reward, while cat images were associated with a timeout. Therefore, monkeys learned to release the bar on green for dog images to obtain the reward and release the bar on red for cat images to avoid the timeout. The neuron data in the category training sessions were not analyzed in this study.

#### 2.1.3 Experimental conditions

The visual stimuli were presented using the Matlab (Mathworks) Psychtoolbox on a Windows operating system (Microsoft). Task control was performed with the REX real-time data-acquisition program adapted to QNX operating system. Four Utah electrode arrays (iridium oxide, Blackrock Microsystems) with 96 electrodes were implanted in Monkey X; one in area TEO, and the others in anterior, middle, and posterior parts of area TE. Three Utah electrode arrays were implanted in the anterior, middle, and posterior parts of area TE in Monkey R. Three arrays were implanted in Monkey L; one in area TEO, and the others in anterior and posterior parts of area TE. Percentage of electrodes with neuronal activities analyzed in Monkeys X, R, and L are shown in Tables 1–3, respectively. The locations of each array are shown in Supplementary Figure 1. TEO is ranged rostro-caudally from an imaginary line perpendicular to the superior temporal sulcus (STS) and tangent to the inferior occipital sulcus (IOS), to a line 1 cm rostral and parallel to the first, and on the dorsal-ventral axis from the fundus of the STS to the fundus of the occipitotemporal sulcus (OTS). TE is ranged rostrally from the rostral boundary of area TEO to an imaginary line connecting the rostral tip of STS with the rostral tip of AMTS and is bounded dorso-medially by the fundus of the STS and ventro-medially by either the fundus of the OTS (caudally), an imaginary line extending from the rostral tip of OTS to the posterior tip of AMTS, or the medial bank of AMTS (rostrally) (Eldridge et al., 2023).

**Table 1:** Percentage of electrodes with neuronal activities analyzed (Monkey X).

**Monkey X**	**Pre (%)**	**Post (%)**
Anterior	15.6	9.4
Middle	75.0	69.8
Posterior	22.9	25.0
TEO	71.9	76.0

**Table 2:** Percentage of electrodes with neuronal activities analyzed (Monkey R).

**Monkey R**	**Pre (%)**	**Post (%)**
Anterior	25.0	17.7
Middle	39.6	35.4
Posterior	62.5	65.6

**Table 3:** Percentage of electrodes with neuronal activities analyzed (Monkey L).

**Monkey L**	**Pre (%)**	**Post (%)**
Anterior	39.6	53.1
Posterior	19.8	18.8
TEO	58.3	55.2

Before each recording session, units were sorted online for the extracellular signal from each electrode using threshold and time amplitude windows. After each recording session, single units were manually sorted offline using principal component analysis in Offline Sorter software (Plexon Inc., Dallas, USA).

All surgical and experimental procedures were approved by the Animal Care and Use Committee of the National Institute of Advanced Industrial Science and Technology (Japan) and were implemented in accordance with the “Guide for the Care and Use of Laboratory Animals” (eighth ed., National Research Council of the National Academies).

### 2.2 Data analysis

#### 2.2.1 Response of neurons to images

To examine the image responsiveness of neurons, we determined whether their activity increased or decreased significantly during stimulus presentation (paired t-test, *p* < 0.05). Multiple comparison correction was performed using the Benjamini-Hochberg method to control for false discovery rate (Benjamini and Hochberg, 1995). If a neuron's firing rate changed significantly in response to at least one image, the neuron was considered to be visually responsive. The number of trials for each image is 19 (X), 5 (R), and 10 (L) in the pre-category training session, 19 (X), 7 (R), and 10 (L) in the post-category training session.

#### 2.2.2 Population vectors

We constructed population vectors to examine category information encoded by a population of neurons *v*_*i*_ (*i* = 1, ⋯ , 520). The elements of a population vector for an image *i* represent the firing rate of neurons in a time window [*t, t*+Δ*t*]. Therefore, the population vector *v*_*i*_ is a *N*-dimensional vector, where *N* is the number of neurons. The firing rate of neurons was calculated by averaging the number of spikes for an image *i* within time window [*t, t*+Δ*t*] by the number of trials. This vector thus represents the firing state of a population of neurons for an image *i* in time window [*t, t*+Δ*t*].

#### 2.2.3 Logistic regression

Using logistic regression (LoR), we investigated the category information encoded by neural populations in area TEO and TE. We performed a binary classification to determine whether the images presented to monkeys were categorized as cat or dog based on population vectors. The LoR model is represented by the following equation using a feature vector Φ (Bishop, 2006);


(1) $$\begin{array}{cc} y\left(\Phi \right) & =\sigma \left(w^{⊤}\Phi \right) \end{array}$$

(2) $$\begin{array}{cc} \sigma \left(a\right) & =\frac{1}{1+\exp\left(-a\right)} \end{array}$$

where *w* is a weight parameter. For data set ${\left\{\phi_{n},t_{n}\right\}}_{n=1}^{N}$, *t*_*n*_∈{0, 1}, this weight parameter is determined to minimize the following cross entropy errors.


(3) $$\begin{array}{c} E\left(w\right)=-\overset{N}{\underset{n=1}{\sum }}\left\{t_{n}\ln\left(\sigma \left(w^{⊤}\phi \right)\right)+\left(1-t_{n}\right)\ln\left(1-\sigma \left(w^{⊤}\phi \right)\right)\right\} \end{array}$$

The LoR model analysis was performed using the scikit-learn library (Pedregosa et al., 2011).

Accuracy of the LoR model was evaluated by 10-fold cross-validation. To investigate the temporal property of category information encoded by neural populations, we fixed the time window width of the population vector at 100 ms and slid the time window by 1 ms. We chose 100-ms duration because a 50-ms window is too brief to capture the neural activity patterns during image fixation in sufficient detail, while a 200-ms window would be too broad, potentially smoothing over important dynamics. In each time window, the accuracy of the LoR model was evaluated.

#### 2.2.4 Fisher's linear discriminant analysis

To investigate how the neural populations in TEO and TE represent cat/dog category information qualitatively, the population vectors in each area were visualized on a one-dimensional space by Fisher's linear discriminant analysis (LDA) (Fisher, 1936; Fukunaga, 1990; Bishop, 2006). We visualized population vectors such that those for the same category appear close together, whereas vectors for different categories are distant from each other. In LDA, a linear transformation *w* is computed that maximizes the separation between class means and minimizes the variance within each class. Using the within-class variance matrix *S*_*W*_ and the between-class variance matrix *S*_*B*_, the LDA criterion *J*(*w*) is defined as follows:


(4) $$\begin{array}{cc} J\left(w\right) & =\frac{w^{⊤}S_{B}w}{w^{⊤}S_{W}w} \end{array}$$

(5) $$\begin{array}{cc} S_{W} & =\overset{2}{\underset{k=1}{\sum }}\underset{n\in C_{k}}{\sum }\left(x_{n}-m_{k}\right){\left(x_{n}-m_{k}\right)}^{⊤} \end{array}$$

(6) $$\begin{array}{cc} S_{B} & =\left(m_{2}-m_{1}\right){\left(m_{2}-m_{1}\right)}^{⊤} \end{array}$$

*m*_*k*_ represents a mean vector of class $C_{k}$ ($C_{1}$ : Cats, $C_{2}$ : Dogs). Since the norm of the vector *w* does not need to be considered, when constraint $w^{⊤}S_{W}w=1$ is imposed. The vector *w* that maximizes the LDA criterion *J*(*w*) is the eigenvector corresponding to the largest eigenvalue in the following eigenvalue equation.


(7) $$\begin{array}{cc} S_{W}^{-1}S_{B}w & =\lambda w \end{array}$$

$$\begin{array}{cc} \lambda & =\frac{w^{⊤}S_{B}w}{w^{⊤}S_{W}w} \end{array}$$

(8) $$\begin{array}{c} =J\left(w\right) \end{array}$$

Ultimately, a vector ŵ that maximizes the LDA criterion is as follows, and the LDA criterion *J*(ŵ) is proportional to the Mahalanobis distance *D*;


(9) $$\begin{array}{cc} ŵ & \propto S_{W}^{-1}\left(m_{2}-m_{1}\right) \end{array}$$

(10) $$\begin{array}{cc} J\left(ŵ\right) & \propto D^{2} \end{array}$$

(11) $$\begin{array}{cc} D & =\sqrt{\left(m_{2}-m_{1}\right)S_{W}^{-1}\left(m_{2}-m_{1}\right)} \end{array}$$

## 3 Results

### 3.1 Single unit visual responses

In a passive fixation task before category training, 87% of neurons in TEO responded to at least one cat or dog image (95% in Monkey X, and 78% in Monkey L). 61% of neurons in TE responded to at least one image (51% in Monkey X, 69% in Monkey R, and 67% in Monkey L). In a passive fixation task after category training, 89% of neurons in TEO responded to at least one image (99% in Monkey X, and 77% in Monkey L). 58% of neurons in TE responded to at least one image (43% in Monkey X, 77% in Monkey R, and 49% in Monkey L). The proportions of neurons that responded to at least one image in TEO and TE were not significantly different between before and after category training (z-test, *p*>0.05).

Tables 4–6 show the number of neurons that responded only to dog images, only to cat images, to both cat and dog images, and to none of the presented images, both pre- and post-category training. The proportion of cat or dog selective neurons does not seem to change with categorization training. The number of neurons with specific category selectivity is quite limited, making it challenging to draw conclusions about category selectivity based solely on these counts. Therefore, we focused our analysis on the temporal patterns of neural responses.

**Table 4:** Number of neurons that responded to dog or cat images (Monkey X).

**Monkey X**	**TEO pre**	**TEO post**	**TE pre**	**TE post**
Only dog	3	1	8	8
Only cat	1	1	7	5
Both cat and dog	84	94	61	45
Not responsive	5	1	74	77
Total	93	97	150	135
Mean firing rate (spikes/sec)	2.33	3.84	2.04	2.82
Std firing rate (spikes/sec)	7.43	5.03	4.34	5.32
Mean latency (ms)	90.3	75.6	37.7	41.9
Std latency (ms)	81.4	74.0	43.8	48.7

**Table 5:** Number of neurons that responded to dog or cat images (Monkey R).

**Monkey R**	**TE pre**	**TE post**
Only dog	7	9
Only cat	6	4
Both cat and dog	89	92
Not responsive	46	32
Total	148	137
Mean firing rate (spikes/sec)	3.58	3.42
Std firing rate (spikes/sec)	6.50	6.06
Mean latency (ms)	106.9	101.4
Std latency (ms)	88.0	87.8

**Table 6:** Number of neurons that responded to dog or cat images (Monkey L).

**Monkey L**	**TEO pre**	**TEO post**	**TE pre**	**TE post**
Only dog	1	3	2	3
Only cat	3	2	6	8
Both cat and dog	57	51	30	23
Not responsive	17	17	19	35
Total	78	73	57	69
Mean firing rate (spikes/sec)	4.60	4.95	1.31	1.55
Std firing rate (spikes/sec)	7.43	6.69	2.92	3.39
Mean latency (ms)	50.6	42.2	55.6	54.4
Std latency (ms)	47.6	38.5	56.5	59.0

**Figure 1:**
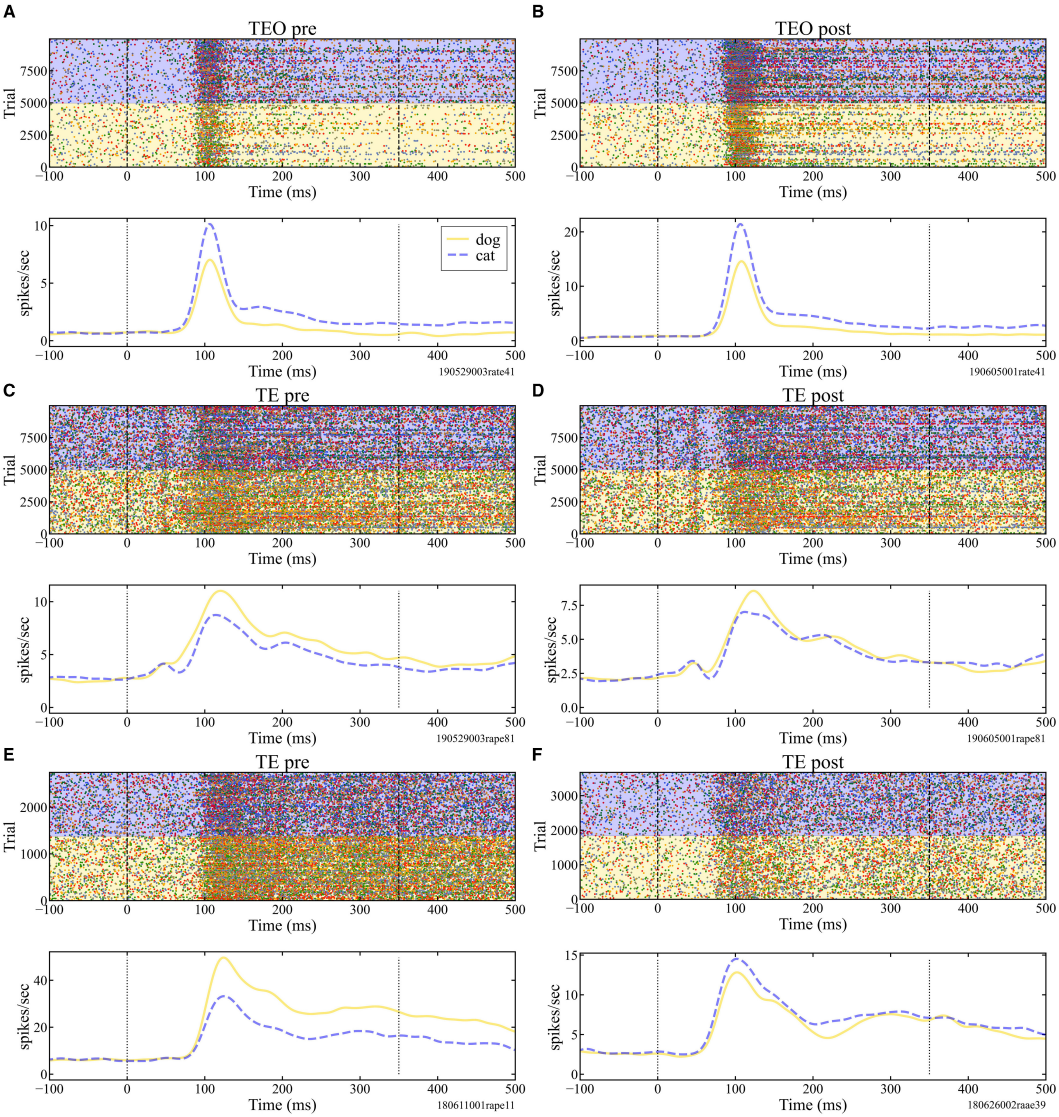
Raster plots (top) and firing rate function (bottom) of area TEO neurons of Monkey X in the pre **(A)** and post **(B)**-session and area TE neurons of Monkey X in pre **(C)**, post **(D)**, Monkey R in pre **(E)** and post **(F)**-session. The neural activities were obtained from the same electrode in pre- and post-session **(A)** vs. **(B)**, **(C)** vs. **(D)**, **(E)** vs. **(F)**. The response examples (top) indicate time in milliseconds (ms) on the horizontal axis and stimulus presentation trials on the vertical axis. Time 0 ms indicates a stimulus onset. Yellow regions mark the responses to dog images, while blue regions indicate responses to cat images. The firing rate functions (bottom) were estimated using kernel density estimation with a Gaussian kernel, with bandwidth fixed at 10 ms. In these plots, the horizontal axis represents time, while the vertical axis represents firing rate per unit of time.

Raster plots and firing rate function of representative single neurons that responded to both cat and dog images are shown in Figure 1 (Figures of neurons that responded only to cat or only to dog images are shown in Supplementary Figures 2, 3). The firing rate of neurons in TEO and TE of Monkey X, TE of Monkey R, and TEO and TE of Monkey L wae significantly different between before and after category training (t-test, *p* < 0.05).

**Figure 2:**
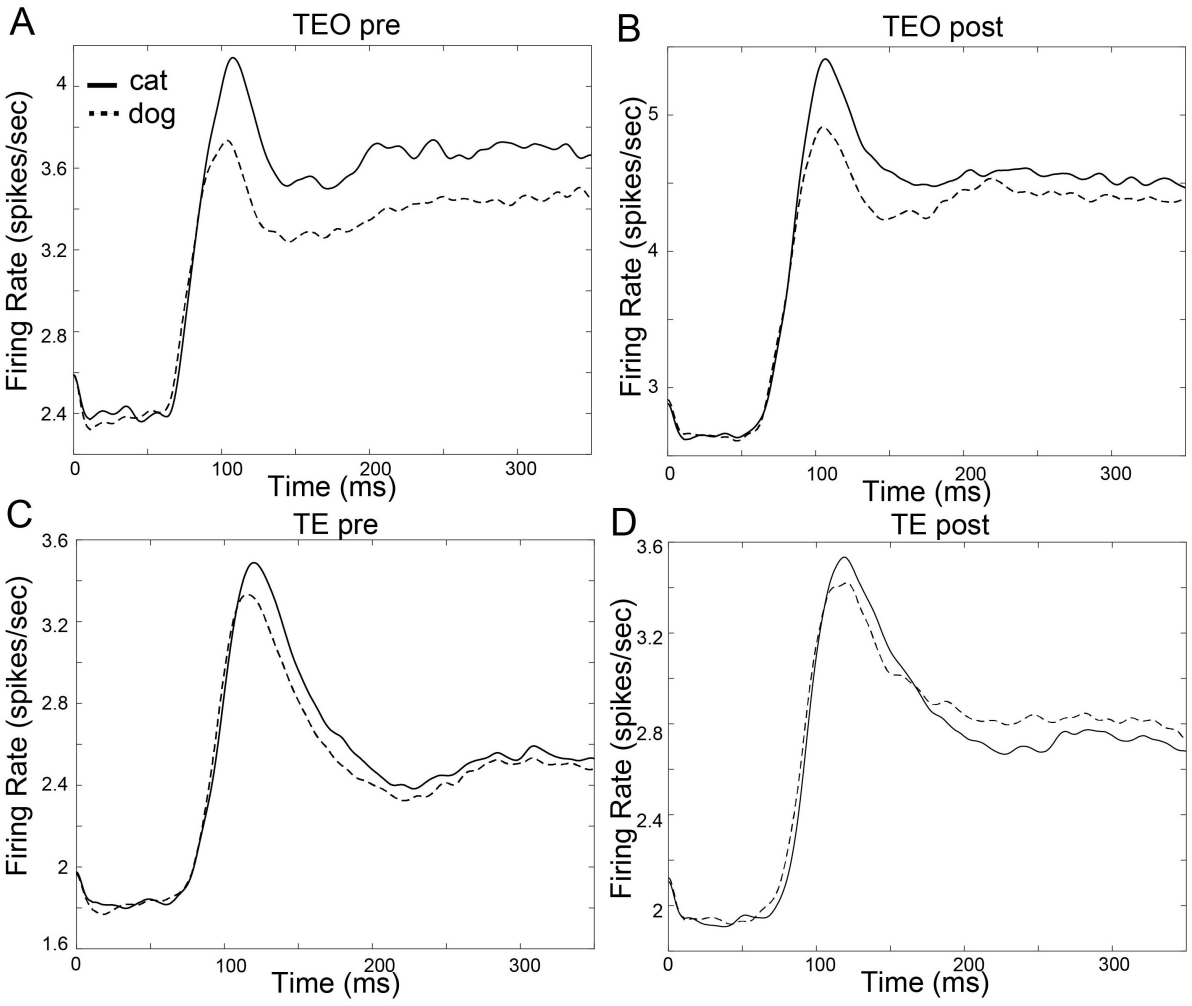
Firing rate function of neuron populations in area TEO and TE. The horizontal axis represents time in milliseconds (ms), while the vertical axis represents the firing rate (spikes/sec). The firing rate functions were estimated using kernel density estimation with a Gaussian kernel, with bandwidth fixed at 10 ms. Time 0 ms indicates a stimulus onset. (**A**): TEO in the pre-category training session (**B**): TEO in the post-category training session (**C**): TE in the pre-category training session (**D**): TE in the post-category training session.

The mean firing rates of population of TEO and TE neurons in the pre- and post-category training sessions are shown in Figure 2. The mean onset latencies are 71.9 ms (SD:70.7 ms, TEO pre), 61.4 ms (SD:63.6 ms, TEO post), 65.4 ms (SD:72.4 ms, TE pre), 68.6 ms (SD:74.6 ms, TE post).

### 3.2 Category encoding—Logistic regression analysis

**Figure 3:**
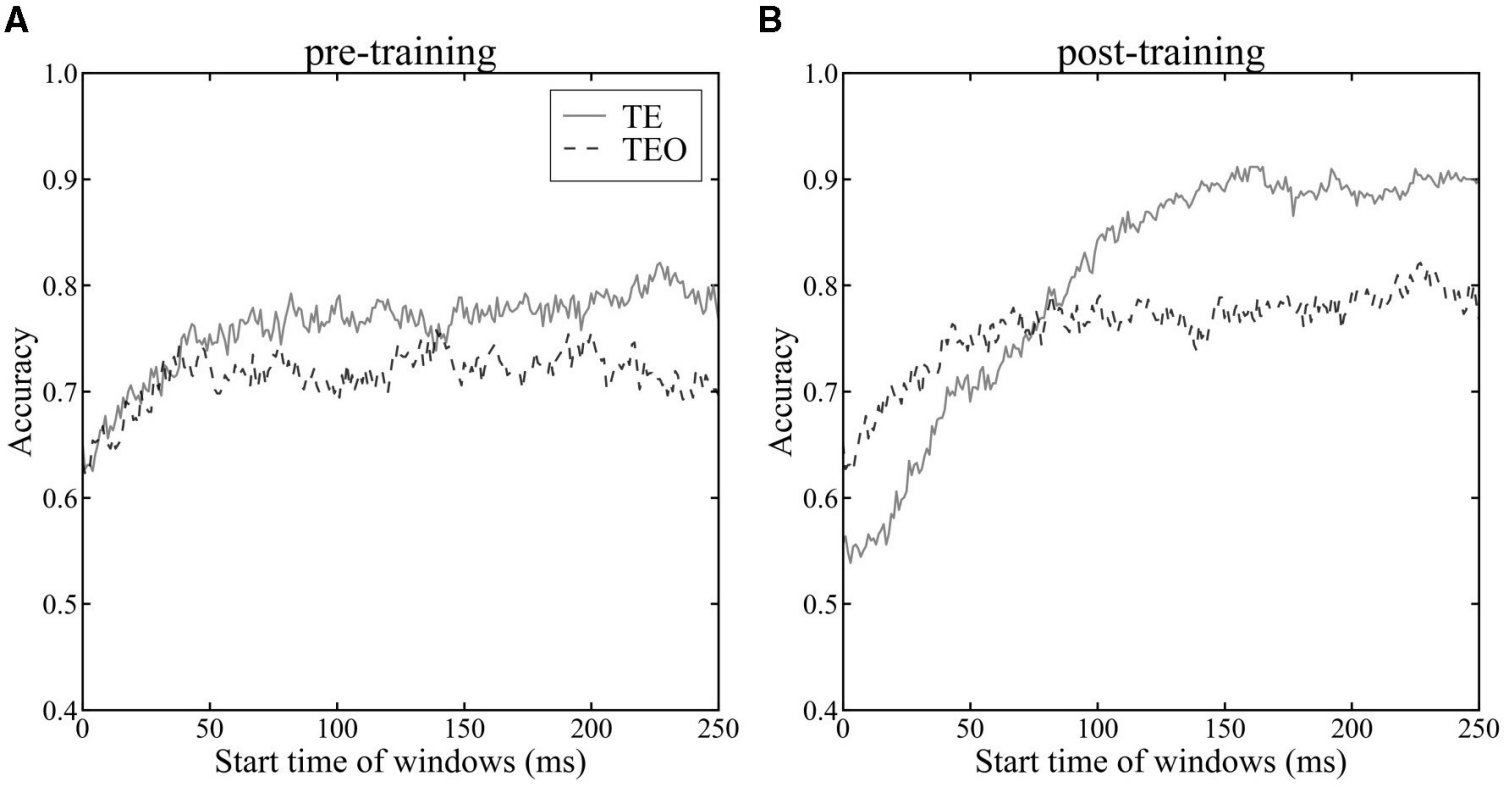
Accuracy of LoR model across time windows. The horizontal axis shows the start time of the time windows, while the vertical axis represents the average accuracy of the LoR model assessed through cross-validation. Time 0 ms indicates a stimulus onset. The dashed line represents the accuracy using the population vector of TEO neurons, while the solid line represents the accuracy using the population vector of TE neurons. Accuracy was evaluated using 10-fold cross-validation. (**A**): pre-category training session (**B**): post-category training session.

We investigated the encoding of category information by neuron populations in areas TEO and TE, measured via the cross-validated accuracy of the LoR model over time (Figure 3). Accuracy of TE neurons significantly increased after the category training (Figure 3A vs. Figure 3B, one-tailed paired *t*-test, *p* < 0.05), indicative of the influence of category learning on model accuracy. After the category training, TE neurons yielded higher accuracy category decoding than that of TEO neurons (Figure 3B, one-tailed paired *t*-test, *p* < 0.05). These findings are in line with previous studies suggesting enhanced category recognition later in the ventral stream (DiCarlo et al., 2012; Hong et al. (2016)). Category encoding in TEO was stable from 100 ms after stimulus onset, whereas for TE improvement in performance was seen up to approximately 150 ms, indicating a difference in temporal dynamics between the two areas. The accuracy in Monkeys X, R, and L is shown in Supplementary Figure 4.

### 3.3 Cat and dog information representation

**Figure 4:**
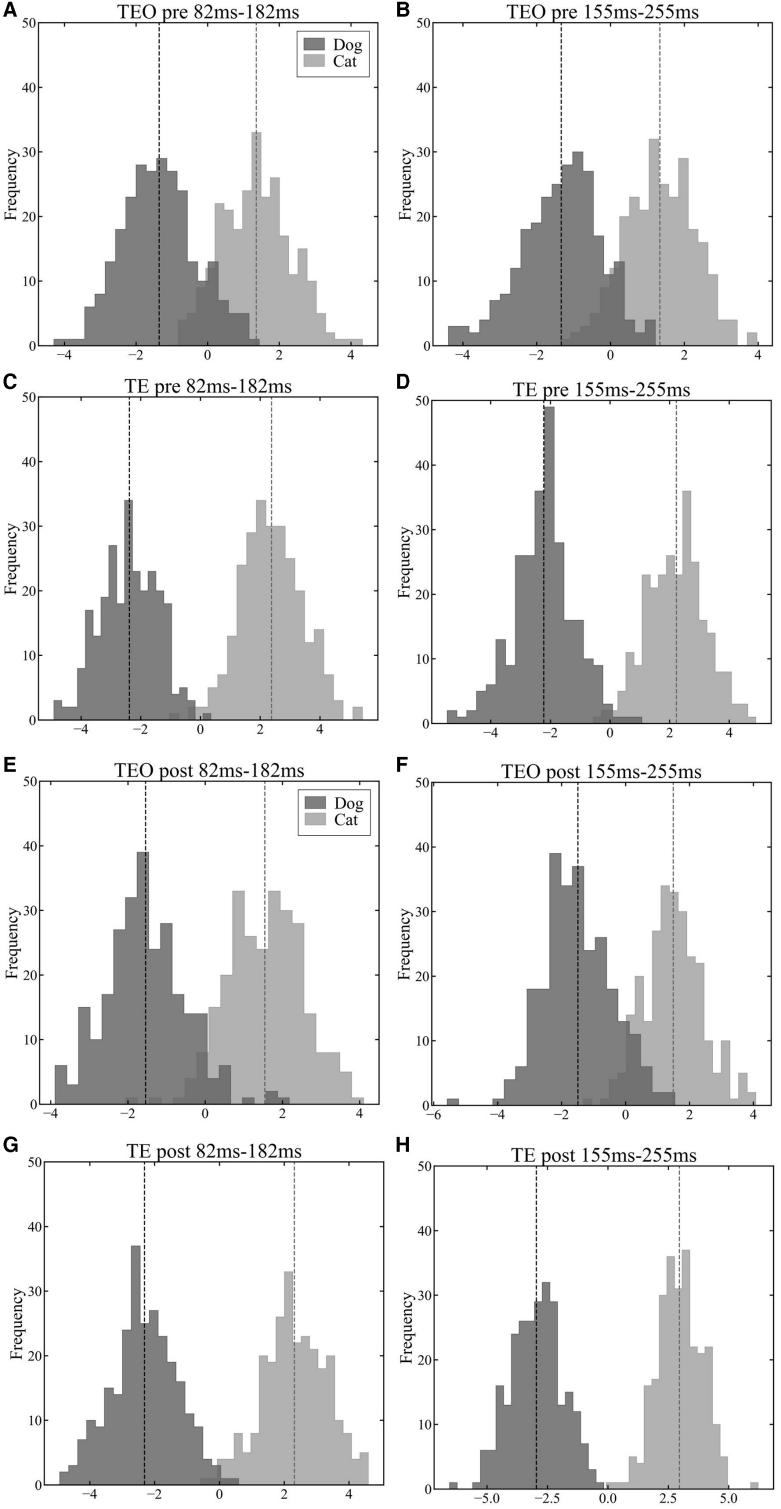
Visualization results of population vectors during the early (82 ms–182 ms) and late (155 ms–255 ms) phases of stimulus presentation in the pre-category training session **(A–D)** and post-category training session **(E–H)** in a first LDA dimension (horizontal axis). The darker histograms represent population vectors corresponding to dog images, and the lighter histograms represent population vectors corresponding to cat images. The vertical dashed lines represent the mean of each distributions. **(A)**: TEO population vectors during early phase of stimulus presentation in the pre-training session. **(B)**: TEO population vectors during late phase of stimulus presentation in the pre-training session. **(C)**: TE population vectors during early phase of stimulus presentation in the pre-training session. **(D)**: TE population vectors during late phase of stimulus presentation in the pre-train session. **(E)**: TEO population vectors during early phase of stimulus presentation in the post-training session. **(F)**: TEO population vectors during late phase of stimulus presentation in the post-training session. **(G)**: TE population vectors during early phase of stimulus presentation in the post-training session. **(H)**: TE population vectors during late phase of stimulus presentation in the post-training session.

To explore the population representation of information in areas TEO and TE, we projected population vectors of each region onto a one-dimensional space using LDA. In a time window from 82 ms to 182 ms after a stimulus onset, the LoR accuracy of TEO neurons reached in a peak while the LoR accuracy of TE neurons reached a peak in a time window from 155 ms to 255 ms. Both in the pre- and post-category training sessions, neither area TEO nor TE neuron populations displayed significant separation between the cat and dog vector distributions in the time window from 82 ms to 182 ms after a stimulus onset (Figure 4A vs. Figure 4E and Figure 4C vs. Figure 4G). However, in the time window from 155 ms to 255 ms, the separation between cat and dog vector distributions of TE neurons in the post-session was larger than in the pre-session (Figure 4D vs. Figure 4H) although the separation of TEO in the post-session was similar to the separation in the pre-session (Figure 4B vs. Figure 4F).

**Figure 5:**
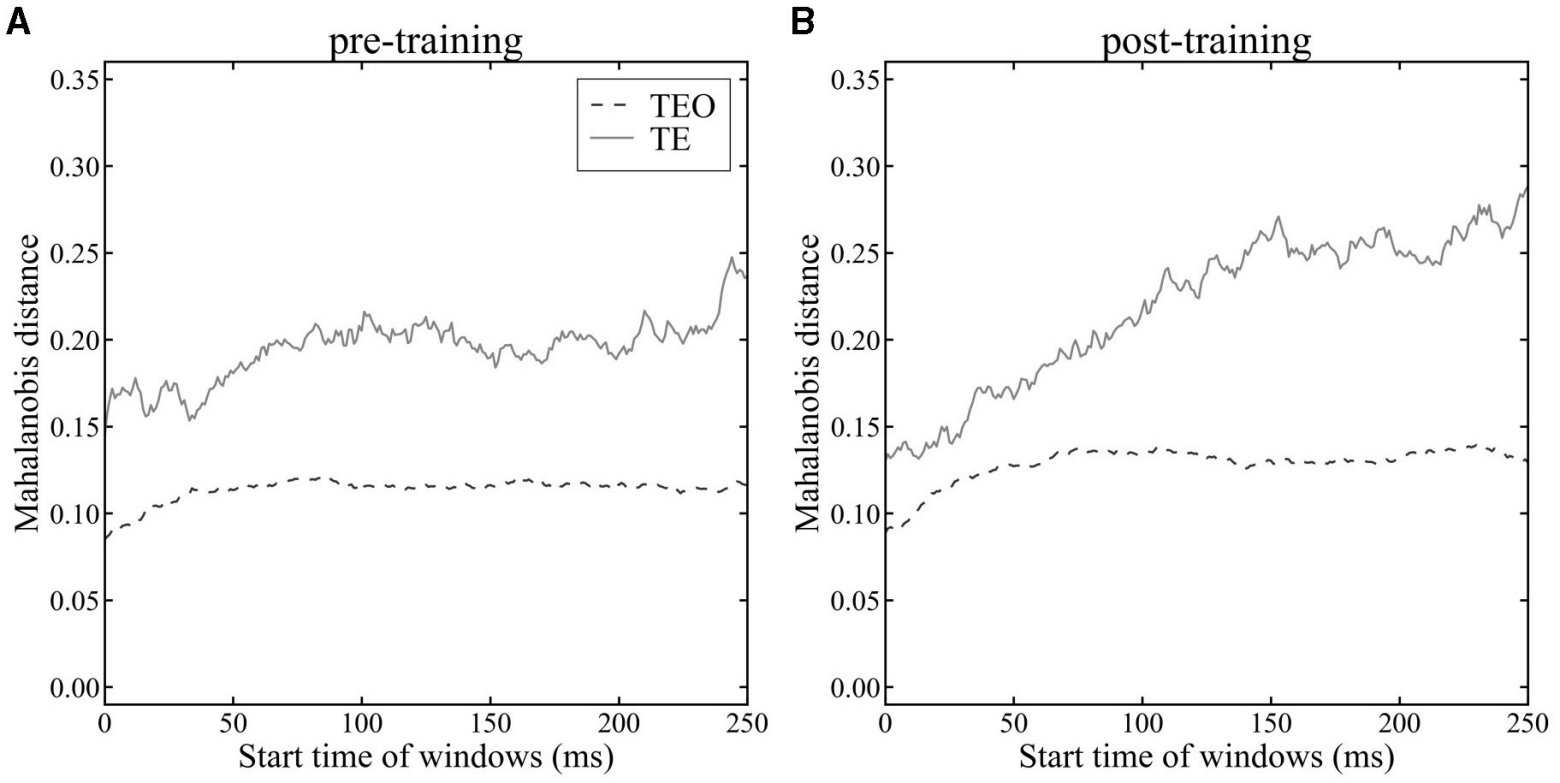
Mahalanobis distance between mean population vectors for cat and dog images in area TEO (dashed line) and TE (solid line). **(A)**: pre-category training session. **(B)**: post-category training session. Time 0 ms indicates a stimulus onset.

To quantitatively investigate the temporal separation of cat and dog image clusters in areas TEO and TE, we calculated the Mahalanobis distance *D* (equation 11) between the mean population vectors during dog stimulus presentations and cat stimulus presentations using a sliding time window (Figure 5). The Mahalanobis distance *D* represents the maximum value of the LDA criterion *J*(w), quantifying the degree of category separation between cat and dog images. Our analysis between pre- and post-category-training sessions using Mahalanobis distance revealed that population vectors in both areas TEO and TE exhibit larger distances in the post-training sessions compared to the pre-training sessions (Figure 5A vs. Figure 5B). The Mahalanobis distance is consistently greater in area TE than in area TEO in both pre- and post-category-training sessions. In area TEO, the distance either stabilizes or shows a decreasing trend starting approximately 100 ms after an image presentation. The mean onset latencies of TE and TEO neurons were less than 100 ms. Therefore, we compared the Mahalanobis distances in the 0–100 ms time window. There was no significant difference in the pre- and post-category-training session (Mann–Whitney U-test, *p*>0.05). Conversely, in area TE, there is a trend of increasing Mahalanobis distance during the image presentation. The Mahalanobis distances calculated from the 250–350 ms time window in the pre- or post-category training session were significantly larger than those from the 0–100 ms time windows (Mann-Whitney U-test, *p* < 0.05). These results quantitatively indicate that in area TEO, there is little temporal change in the separation between cat and dog categories, whereas in area TE, temporal category separation improves within a trial. The Mahalanobis distance in Monkey X, R, and L is shown in Supplementary Figure 5, and the Mahalanobis distance before a stimulus onset is shown in Supplementary Figure 6.

Visualizing population vectors and computing the Mahalanobis distance *D* (Equation 11) between average population vectors during cat and dog stimulus presentations revealed temporal properties of category discrimination consistent with the temporal trends seen in LoR model analyses. In the analysis with LoR, there was not clear difference between TE and TEO for pre-category training session (Figure 3A), whereas there was a clear difference between TE and TEO in analysis using Mahalanobis distance (Figure 5). The LoR model accuracy reflects category encoding performance, while the Mahalanobis distance measures the distance between two categories. These two methods had different roles as discussed in the discussion section. Therefore, this difference looks reasonable.

The difference in Mahalanobis distance between TE and TEO was found even in the very early window (0–100 ms). The mean onset latencies of TE and TEO neurons were less than 100 ms. Therefore, the Mahalanobis distances in 100-ms time window changed even in early time window (0–100 ms). Before stimulus onset (in −200 to 0 ms, Supplementary Figure 6), the differences of Mahalanobis distances between TE and TEO did not change a lot, almost flat. The Mahalanobis distance in TE or TEO after stimulus onset was larger than before a stimulus onset.

## 4 Discussion

To investigate the influence of category training experience on category encoding in neural populations in IT cortex, we recorded neuronal activity in area TE from three monkeys, and in TEO from two of the three, during a passive fixation task. We analyzed the category information encoded by their neuronal populations using three analysis methods (LoR, LDA, and the Mahalanobis distance). In LoR, we calculated encoding accuracy for cat and dog categories (Figure 3). LDA determined a low-dimensional space with the largest Mahalanobis distances between the cat and dog categories. Each population vector for each image was shown in one-dimensional LDA space in two different time windows to visualize the population vectors for individual cat and dog images (Figure 4). To investigate the detail temporal change of the separation between cat and dog categories, we showed Mahalanobis distance in each time window (Figure 4). Therefore, these three measures have different roles. We obtained consistent results across the three methods of analysis, suggesting that our observations regarding the temporal properties of category information processing differences between areas TEO and TE are robust. The LoR model of TEO neurons produced increased category discrimination performance during the initial phase of stimulus presentation and then remained stable. Conversely, the LoR model of TE neuorons continued to show improvement in category discrimination performance into later time windows than did TEO. Additionally, by visualizing population vectors with LDA, we compared category representations of cats and dogs in areas TE and TEO. Comparing visualization results from the early and late phases of stimulus presentation, we qualitatively confirmed that category representations in area TE become more distinctly separated during stimulus presentation than do representations in area TEO. The temporal characteristics of the respective regions were quantitatively evaluated by calculating the Mahalanobis distance between populations in both areas. The temporal properties of the information represented by the neural populations in the two areas, within a trial, were consisted with the temporal trends in category discrimination reported using the LoR model analysis—little change in category information during the trial in area TEO, but increased category information in area TE during the trial.

In the post-category training session, the neuronal populations in area TE exhibited superior categorization capabilities over area TEO (Figure 3B). Previous studies have shown that the category discrimination capabilities of neuronal populations improve along the pathway from V1 to IT (DiCarlo et al., 2012; Hong et al., 2016). The fact that category discrimination abilities are higher in TE than TEO aligns with the observation that category information for complex objects can be more easily decoded with a linear decoder at later stages of the ventral stream. The category discriminative ability of TEO neuronal populations improved during the initial phase of stimulus presentation and then remained stable. The initial improvement could be considered a reflection of the latency in image responses. However, category discrimination capabilities in populations of TE neurons continued to improve at later time windows than those in TEO. There was no significant difference between the onset latencies of TEO and TE neurons (Figure 2, t-test, *p*>0.05). Hence, the difference in accuracy during the trial between the LoR models of TEO and TE is likely not due to differences in onset latency, but rather due to differences in the temporal nature of category information processing. Most electrophysiological studies of category information processing have analyzed either TE alone or IT's encoding of category information (Sugase et al., 1999; Matsumoto et al., 2005; Kiani et al., 2007; Meyers et al., 2008; Kriegeskorte et al., 2008; Dehaqani et al., 2016; Hong et al., 2016). However, as the analysis using the LoR model suggests, there are differences in categorization capabilities and their temporal properties between areas TEO and TE, making it beneficial to distinguish and analyze them separately.

Our study found that the time at which neuronal populations in area TE exhibited the highest categorization capability in the post-category training session was between 155 ms and 255 ms following stimulus presentation. This indicates the amount of category information for cats and dogs encoded by the neuronal populations in TE peaked approximately 155 ms after an image was presented. According to Sugase et al. (1999), the peak for category information for human vs. monkey faces in TE neurons occurs approximately 100 ms after stimulus presentation. The difference in the timing of these peaks may reflect differences in the image sets presented to the monkeys. Processing category information for cats and dogs is more challenging than that for primate faces (Matsumoto et al., 2016). Additionally, the images of cats and dogs presented in this study were associated with waiting time (cats) or a reward (dogs) through the category training task. Therefore, after the category training, the amount of category information may increase due to feedback from higher brain areas, e.g., perirhinal or prefrontal cortex. Such feedback might also contribute to the observed differences in the timing of the peak.

As shown by the visualization of population vectors through LDA, histograms corresponding to cat and dog distributions overlap to the same extent in early (82 ms–182 ms) and late (155 ms–255 ms) time windows for neuronal populations in area TEO (Figures 4A, B, E, F). In contrast, for TE neuronal populations, distributions were distinctly separated in the later window compared to the earlier time window (Figures 4C, D, G, H). Furthermore, the Mahalanobis distance *D* (Equation 11) (Figure 5) between population vector means for cat and dog images increased over time in area TE, while it remained constant in area TEO. This shows that the separation of category representations within a trial is progressing in area TE. Such separation enhances the LoR model's ability to discriminate categories; thus these results are consistent. Moreover, our data for TE neuronal populations showed a larger Mahalanobis distance immediately following a stimulus presentation in the pre-category training session than in the post-training session. The accuracy rates of LoR models trained with TE populations also showed greater distinction in the time window immediately after a stimulus onset in the pre-category training session than in the post (Figures 3A, B), reflecting the relationship between Mahalanobis distances.

Neurons in perirhinal cortex responds the association between visual images and reward (Mogami and Tanaka, 2006; Ohyama et al., 2012). During the category training, monkeys were rewarded for releasing a bar when the color of a square changed from red to green in dog-presentation trials. Conversely, in cat-presentation trials, they had to wait for the next trial if they released a bar during a green period. This task learning may have altered the connectivity state between TE and other areas, such as the perirhinal cortex, and influenced the temporal changes in TE's categorization capabilities based on feedback from these regions.

Another explanation for the temporal changes in categorization during a stimulus presentation in TE is that the representation of category information encoded by TE neuronal populations changes over time. Studies have shown that individual neurons and neuronal populations in TE encode different representations of information over time (Sugase et al., 1999; Matsumoto et al., 2005). These studies imply a temporal shift in information representation from coarse to fine classifications in TE neurons, but the detailed comparison between this study and the corase-to-fine categorization will be discussed in a future work.

The LoR accuracy of TEO neuronal populations improved within 50 ms after a stimulus presentation, which is consistent with the latency of visual information arriving in this region and then exhibited a steady trend during stimulus presentation. Furthermore, there was no progression during a trial in the separation of cat and dog distributions obtained by visualizing population vectors. Compared with the analysis of TE neurons, our analysis suggests that there is little temporal change in category information representation among TEO neuronal populations. However, since only cat and dog categories were presented in this study, we could not examine in detail the temporal changes in category representation in the two areas. By visualizing population vectors, when presenting an image set with a hierarchical category structure, as demonstrated in previous studies (Sugase et al., 1999; Matsumoto et al., 2005; Dehaqani et al., 2016), in areas TEO and TE (using methods such as principal component analysis or LDA), and analyzing the temporal changes of these visualized vectors, significant insights can be gained into the temporal changes in category representation in TEO and TE neurons. This will be discussed in a future work.

## 5 Conclusion

We investigated the influence of experience on category information processing in areas TEO and TE. A previous study by Pearl et al. characterized the effect of experience on category representation in TE neurons (Pearl et al. (2024)). Our analyses provide additional insight into the temporal characteristics of category representation, and contrast category encoding, in TE neurons with that in TEO neurons. We thereby demonstrate that area TE neurons exhibit sufficient plasticity after category training.

## Data Availability

The raw data supporting the conclusions of this article will be made available by the authors, without undue reservation.
