# Supplementary material for: Differences in category information processing between areas TEO and TE of the macaque
Source: Front Behav Neurosci. 2025 Jan 29;18:1449097. doi: 10.3389/fnbeh.2024.1449097 (PMC11814174; doi:10.3389/fnbeh.2024.1449097)
Supplement: Supplementary file 1 [file Data_Sheet_1.pdf]

Supplementary Material

1 SUPPLEMENTARY FIGURES

1.1 Locations of recording sites in three monkeys

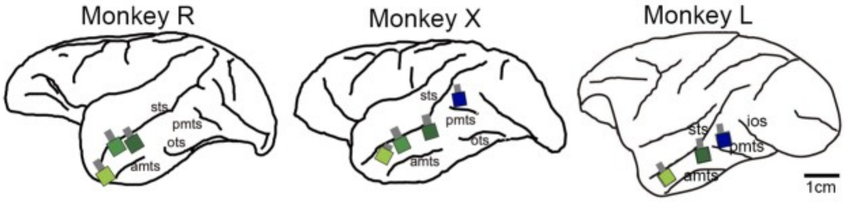

Figure 1. Utah array locations in three monkeys

1.2 Single unit response only to dog or cat images

Figure 2a.

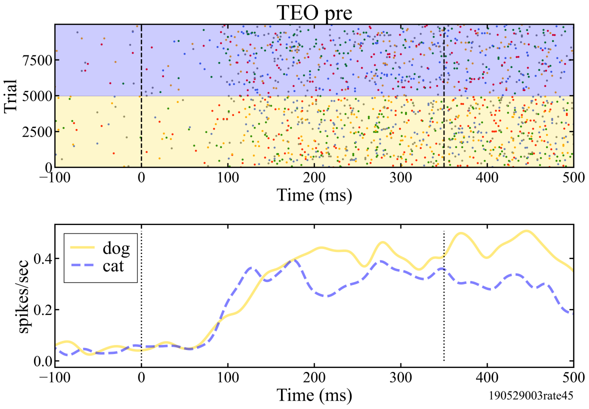

Figure 2b.

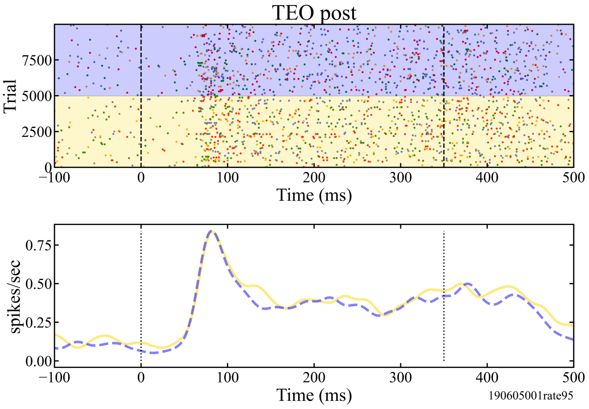

Figure 2c.

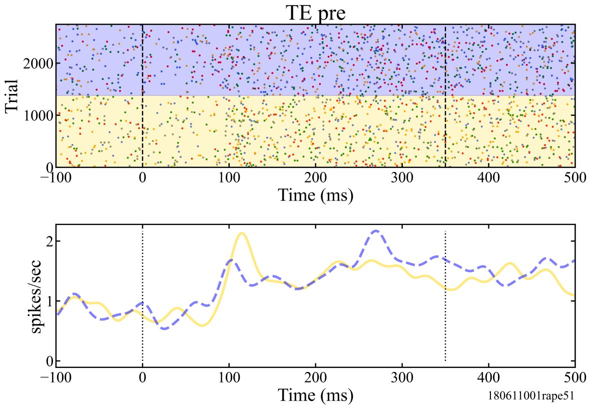

Figure 2d.

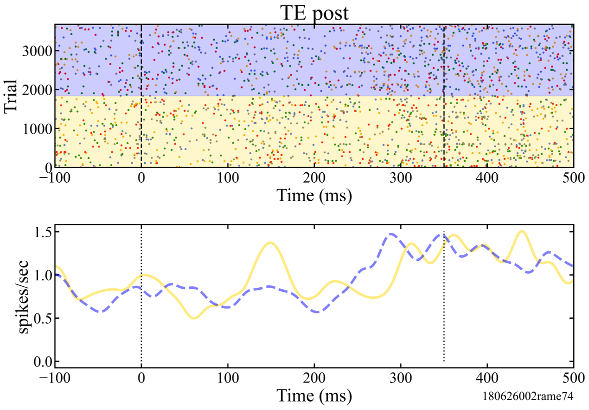

Figure 2. Raster plots and firing rate function of neurons that responded only to dog images, in area TEO of Monkey X in the pre (2a) and post (2b) session, and area TE of Monkey R in pre (2c) and post (2d) session. Figure 2 illustrate examples of responses (top) and firing rate functions (bottom) of neurons in areas TEO and TE, pre and post session. The response examples (Figure 2 top) indicate time in milliseconds (ms) on the horizontal axis and stimulus presentation trials on the vertical axis. Yellow regions mark the responses to dog images, while blue regions indicate responses to cat images. The firing rate functions (Figure 2 bottom) were estimated using kernel density estimation with a Gaussian kernel, with bandwidth fixed at 10 ms. In these plots, the horizontal axis represents time, while the vertical axis represents firing rate per unit of time.

Figure 3a.

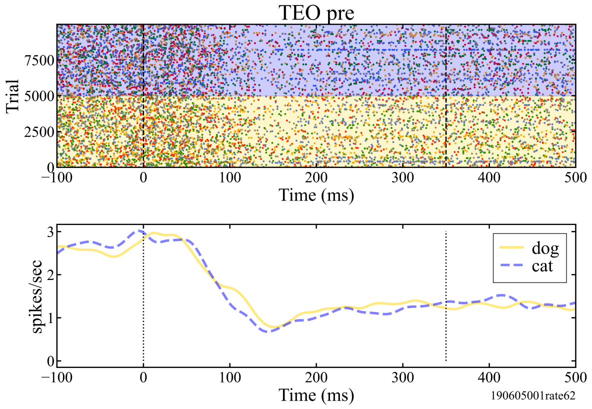

Figure 3b.

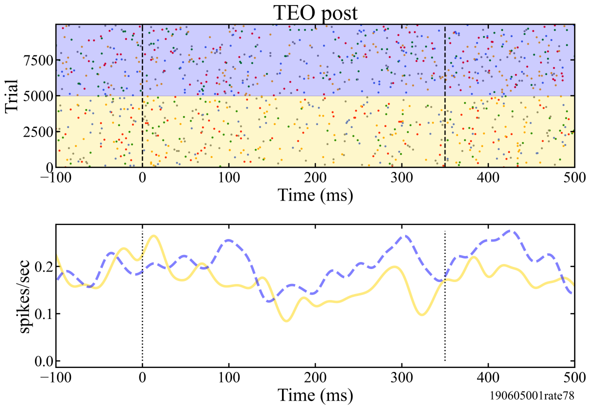

Figure 3c.

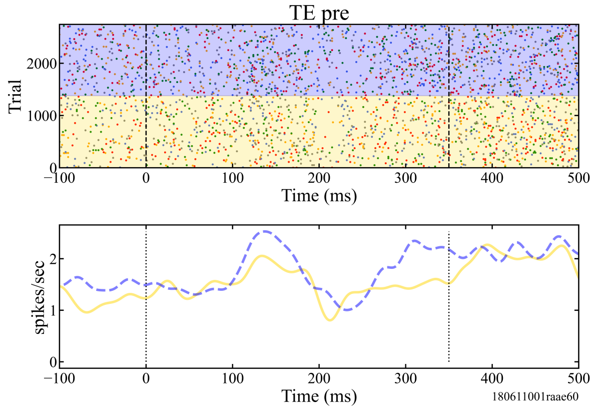

Figure 3d.

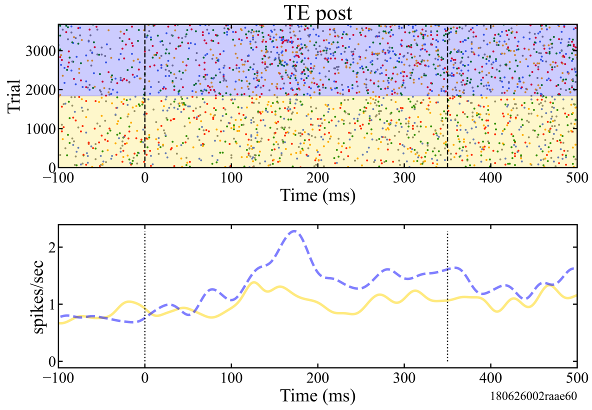

Figure 3. Raster plots and firing rate function of neurons that responded only to cat images, in area TEO of Monkey X in the pre (3a) and post (3b) session, and area TE of Monkey R in pre (3c) and post (3d) session. Figure 3 illustrate examples of responses (top) and firing rate functions (bottom) of neurons in areas TEO and TE, pre and post session. The response examples (Figure 3 top) indicate time in milliseconds (ms) on the horizontal axis and stimulus presentation trials on the vertical axis. Yellow regions mark the responses to dog images, while blue regions indicate responses to cat images. The firing rate functions (Figure 3 bottom) were estimated using kernel density estimation with a Gaussian kernel, with bandwidth fixed at 10 ms. In these plots, the horizontal axis represents time, while the vertical axis represents firing rate per unit of time.

1.3 Category encoding by neurons in each monkey - logistic regression analysis

Figure 4a.

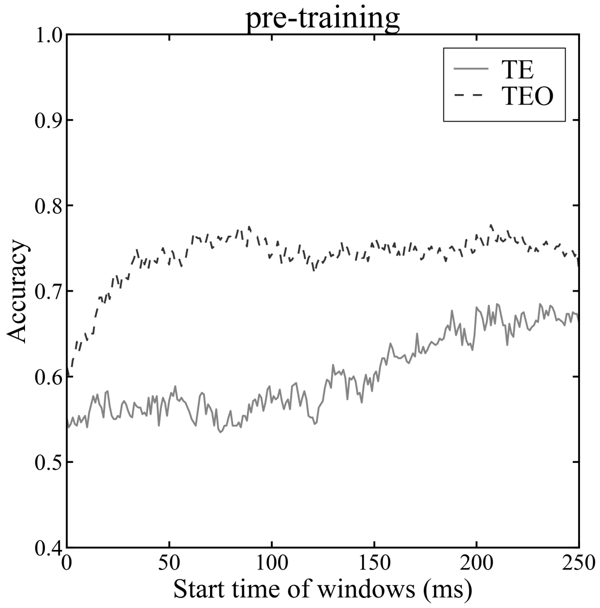

Figure 4b.

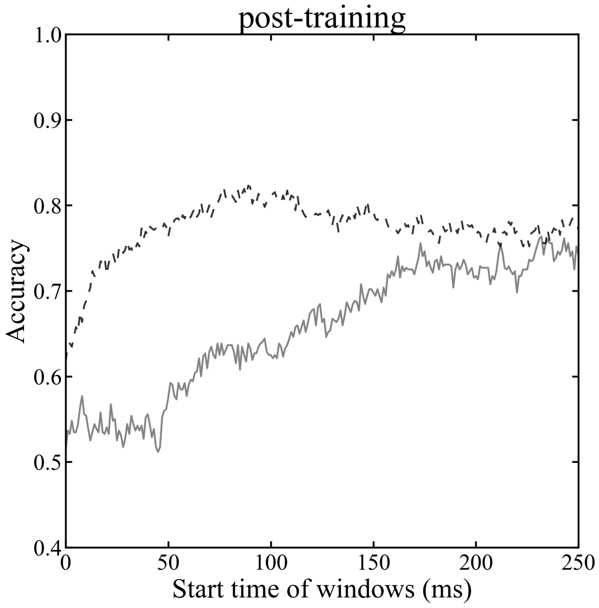

Figure 4c.

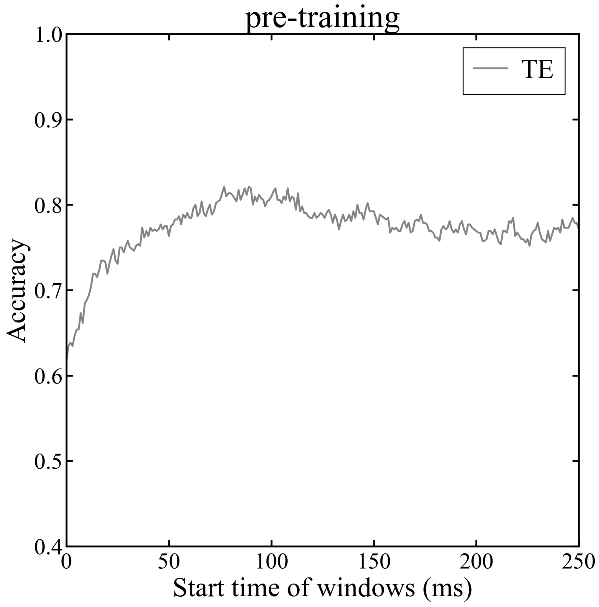

Figure 4d.

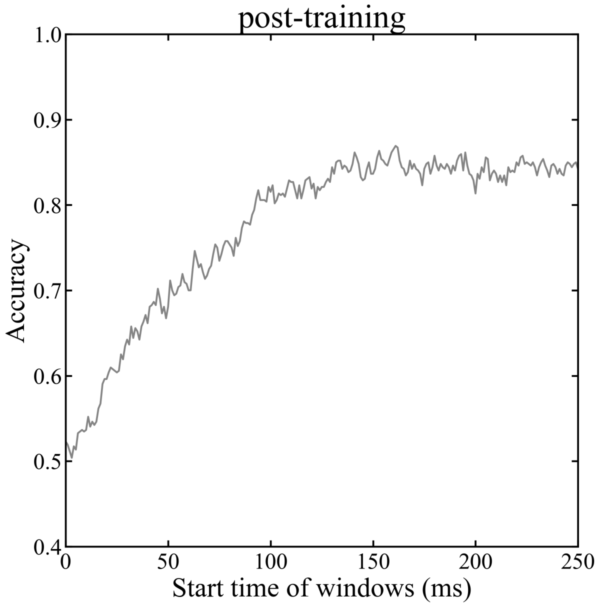

Figure 4.

Figure 4e.

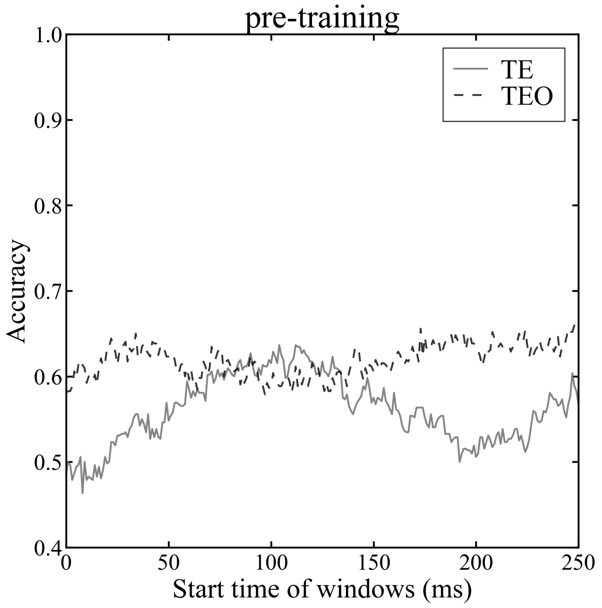

Figure 4f.

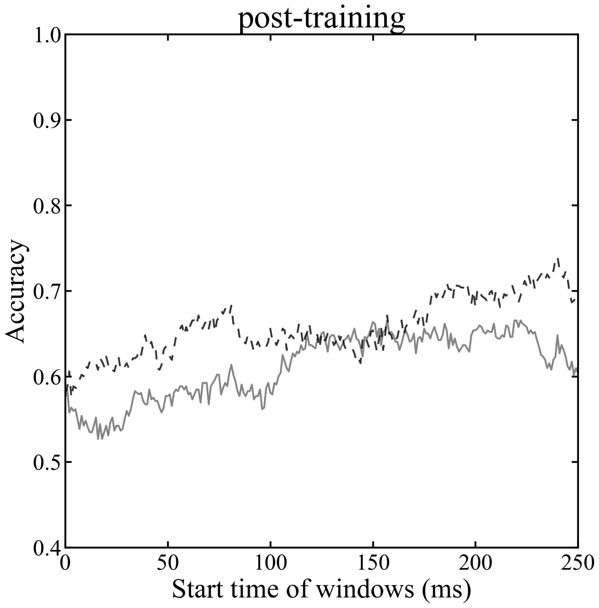

Figure 4. Accuracy of LoR model across time windows. The horizontal axis shows the start time of the time windows, while the vertical axis represents the average accuracy of the LoR model assessed through cross-validation. The dashed line represents the accuracy using the population vector of TEO neurons in Monkey X or L, while the solid line represents the accuracy using the population vector of TE neurons in Monkey X or L. Accuracy were evaluated using 10-fold cross validation. (**4a**): Monkey X (pre-training session) (**4b**): Monkey X (post-training session) (**4c**): Monkey R (pre-training session) (**4d**): Monkey R (post-training session) (**4e**): Monkey L (pre-training session) (**4f**): Monkey L (post-training session)

1.4 Cat and Dog information representation in each monkey

Figure 5a.

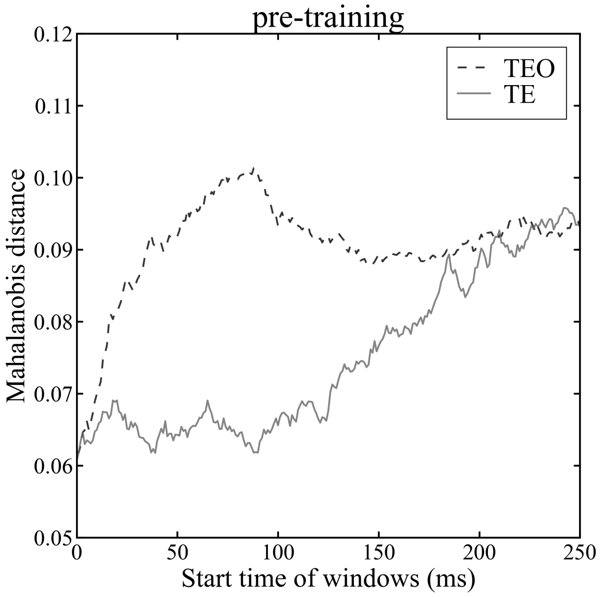

Figure 5b.

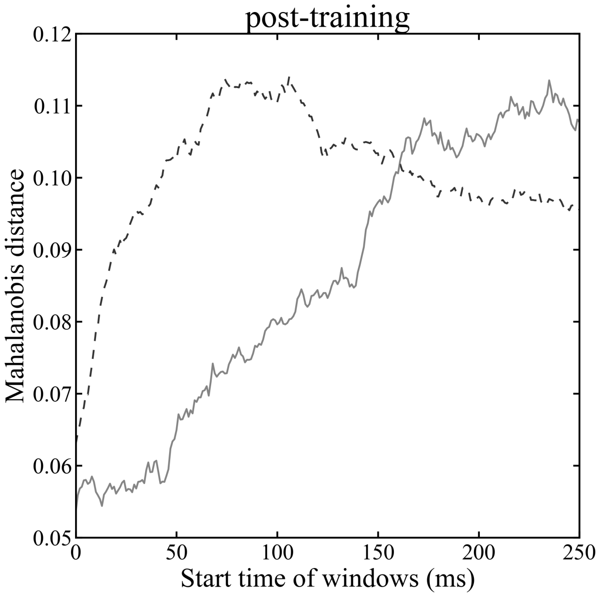

Figure 5c.

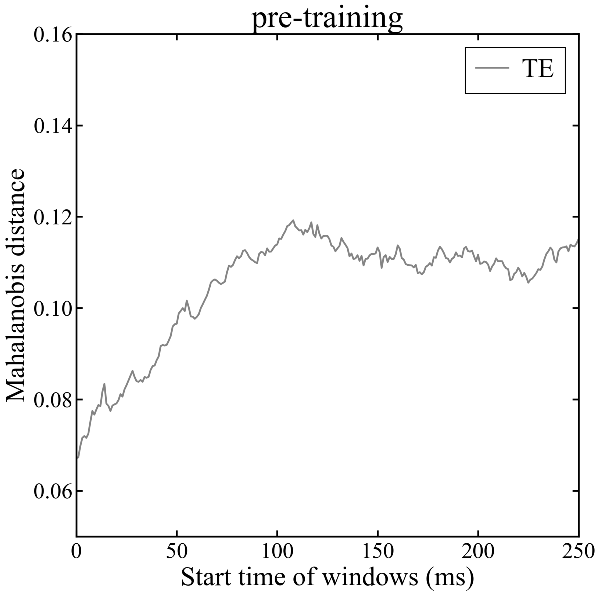

Figure 5d.

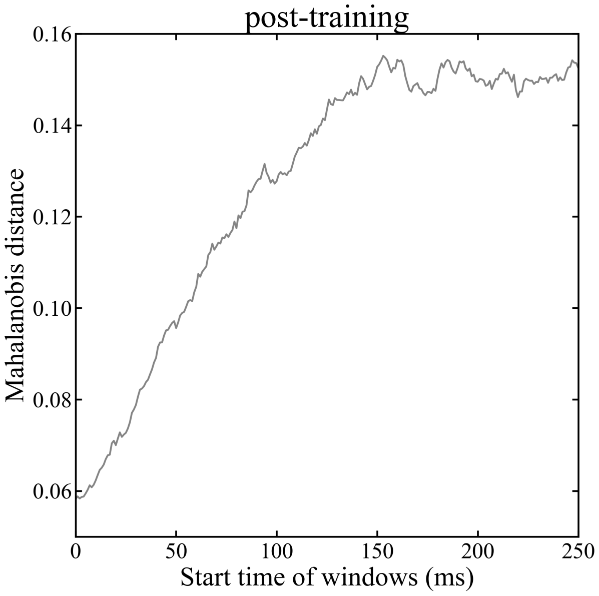

Figure 5.

Figure 5e.

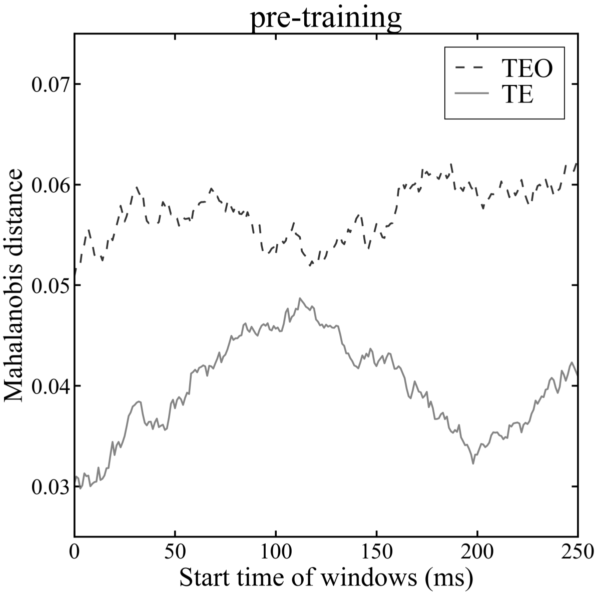

Figure 5f.

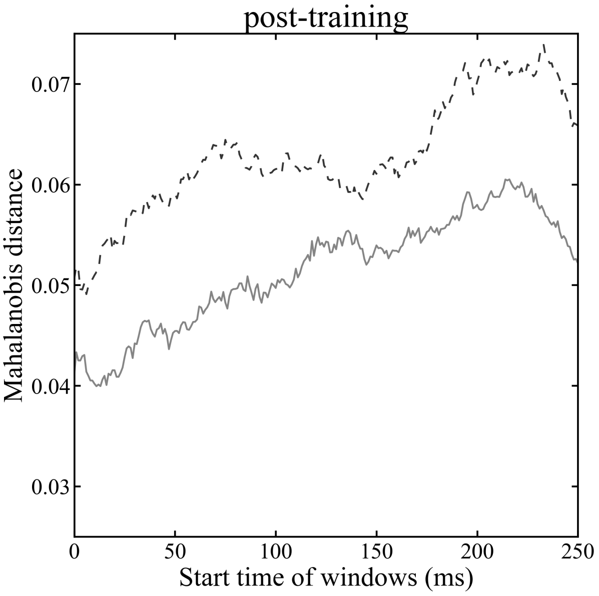

Figure 5. The Mahalanobis distance between mean population vectors for cat and dog images in area TEO (dashed line) and TE (solid line). **(5a)**: Monkey X (pre-training session) **(5b)**: Monkey X (post-training session) **(5c)**: Monkey R (pre-training session) **(5d)**: Monkey R (post-training session) **(5e)**: Monkey L (pre-training session) **(5f)**: Monkey L (post-training session)

1.5 Category information representation before image presentation

Figure 6a.

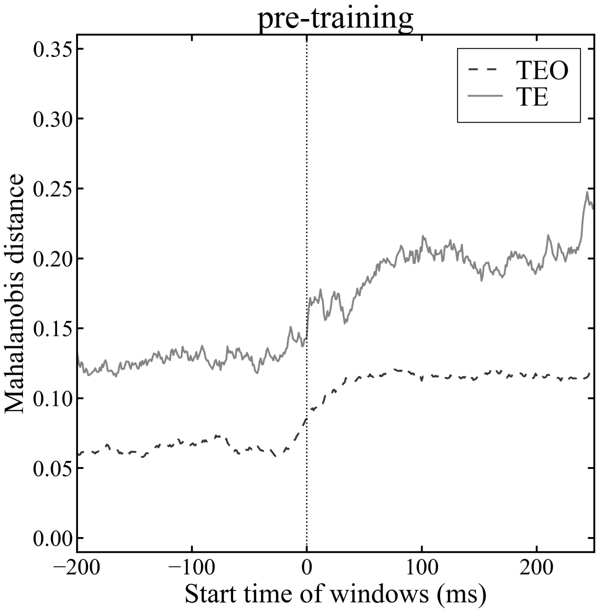

Figure 6b.

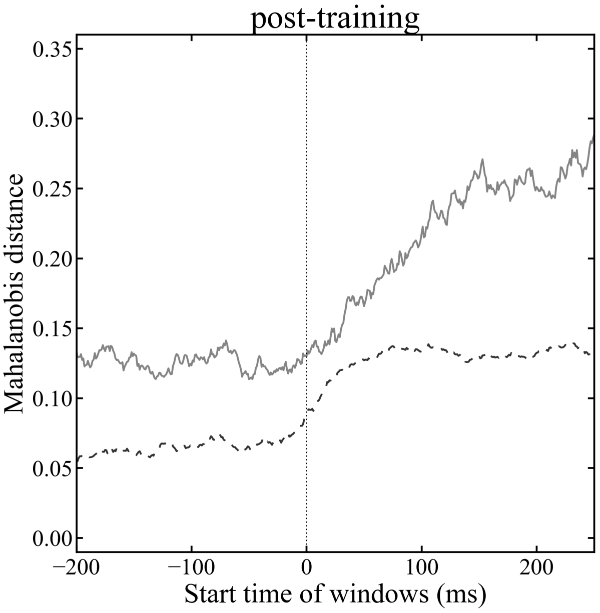

Figure 6. The Mahalanobis distance between mean population vectors for cat and dog images in area TEO (dashed line) and TE (solid line). Time 0 ms indicates the onset of the image presentation. **(6a)**: pre-training session **(6b)**: post-training session
